# A robust system of hybridogenesis that increases genetic variability and promotes evolutionary succession in greenlings (Teleostei: Hexagrammidae, genus *Hexagrammos*): Regeneration of a new hemiclonal lineage

**DOI:** 10.1371/journal.pone.0304772

**Published:** 2024-06-03

**Authors:** Shota Suzuki, Shunsuke Yoshida, Misaki Aratani, Motoko R. Kimura-Kawaguchi, Hiroyuki Munehara

**Affiliations:** 1 Graduate School of Environmental Science, Hokkaido University, Sapporo, Japan; 2 Usujiri Fisheries Station, Field Science Center for Northern Biosphere, Hokkaido University, Hakodate, Japan; Complutense University of Madrid: Universidad Complutense de Madrid, SPAIN

## Abstract

Unisexual hybrids that reproduce either clonally or hemiclonally are considered to be evolutionarily short-lived as they lack the ability to reduce deleterious mutations and increase genetic diversity. In the greenling (Teleostei: Hexagrammidae, genus *Hexagrammos*), unisexual hybrids that produce haploid eggs containing only the *H*. *octogrammus* (maternal species) genome generate hemiclonal offspring by fertilization with haploid sperm of *H*. *agrammus* (paternal species). When hemiclonal hybrids are backcrossed to a male of the maternal species, the offspring (BC-*Hoc*) are phenotypically similar to the maternal species and produce recombinant gametes through conventional meiosis. BC-*Hoc* (recombinant generation) individuals referred to as carriers harbor the genetic factor for hybridogenesis, thereby facilitating the production of new hemiclonal lineages through hybridization. Previous studies based on field research have suggested that the carriers produced by two-way backcrossing (mating pattern in which hemiclonal hybrids are backcrossed with both parental species) may overcome the evolutionary dead end imposed by the lack of recombination. The present study verified this hypothesis by regenerating a newly hemiclonal lineage through artificial hybridization. To clarify the genetic mode of hybrids produced by crosses between BC-*Hoc* and *Hag*, mature eggs were obtained from 16 individuals and fertilized with either *Hag* or *Hoc* sperm. Hybridogenesis was confirmed in one of the 16 individuals. Based on the low occurrence rate, these findings suggest that hemiclonal lineages can be regenerated, and that the hemiclonal factors are likely distributed across multiple genes on different chromosomes. The findings provide important evidence for the retention of a robust system for increasing genetic variability and maintaining evolutionary succession in unisexual hybrids that reproduce hemiclonally.

## Introduction

Interspecific hybridization is observed frequently in fish [1, 2]. While interspecific hybrids are often infertile due to pairing incompatibilities between homologous chromosomes, some hybrid lineages maintain their populations by switching between bisexual and unisexual modes of reproduction by employing mechanisms such as clonal reproduction (parthenogenesis and gynogenesis) or hemiclonal reproduction (hybridogenesis) [3–7]. In clonal reproduction, the female produces unreduced diploid or triploid eggs, and sperm are used only to trigger embryogenesis [5]. Therefore, the male makes no biological or genetic contribution to the offspring. Conversely, in hemiclonal reproduction (hybridogenesis), the paternal genomes also play a role in somatic growth during embryogenesis after fertilization, which means that the offspring retain both the maternal and paternal genomes in their somatic cells. However, during oogenesis within the gonadal tissue, one parental genome, typically the paternal genome, is excluded from germ cells, which culminates in the production of haploid eggs that carry only the other parental genome [8–10].

Without recombination, unisexual hybrids are expected to become extinct in 10,000 to 100,000 generations because they accumulate deleterious mutations in their genomes and genetic diversity is limited [11–13]. There are several considerations regarding the mechanisms by which unisexual organisms produce genetic variation, such as the partial incorporation of paternal chromosomes and increasing ploidy [3, 14]. However, neither the mechanisms that reduce the extinction risk and break the evolutionary dead end in unisexual vertebrates, nor the conditions for creating new unisexual reproductive lineages are currently well understood [15].

The "balance hypothesis" [16, 17] is a well-known explanation for why unisexual reproduction occurs. According to this hypothesis, unisexual lineages occur in very limited cases, such as when the genetic dissimilarity between two sexual species is extensive enough to disrupt typical gametogenesis, but the hybrids remain viable and sufficiently similar to contribute towards the development of a stable phenotype [16, 17]. However, recent studies have suggested that unisexual reproduction might be caused not only by the extent of the genomic dissimilarity between species, but also by “genetic factors” to induce hybridogenesis [15, 18–20].

**Fig 1:**
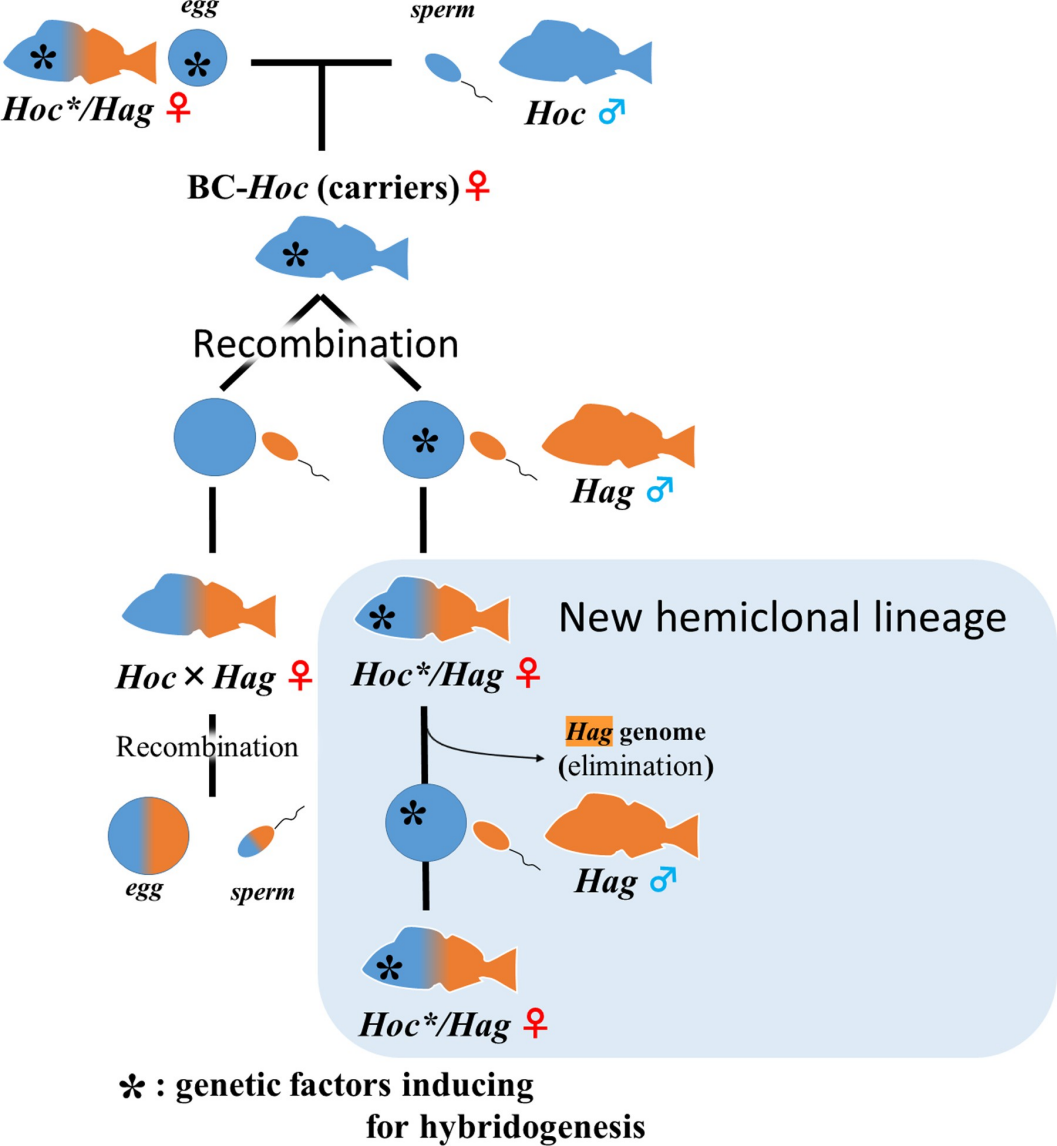
Lineages of the *Hexagrammos* hybrids referred to in the present study. If hybrids form between BC-*Hoc* and *Hag*, then hybridogenesis would be perpetuated by the inheritance of genetic factors that induce hybridogenesis; in the absence of such an inheritance pattern, recombinant reproduction would occur. *Hoc* and *Hag* represent *H*. *octogrammus* and *H*.*agrammus*, respectively. Blue and orange indicate the genomes of *H*. *octogrammus* and *H*. *agrammus*, respectively.

The genus *Hexagrammos* includes six species of greenlings that are widespread in the coastal waters of the North Pacific Ocean [21]. One boreal species, the masked greenling *H*. *octogrammus* (hereafter *Hoc*), and two temperate species, the fat greenling *H*. *otakii* (*Hot*) and the spotty belly greenling *H*. *agrammus* (*Hag*), have sympatric distributions in coastal areas of southern Hokkaido (Japan) and Primorsky Krai (Russia) [22]. The hybrid zone that is inhabited by these three *Hexagrammos* species contains two unisexual hybrids that have been reported to reproduce hemiclonally [19]. These hemiclonal hybrids produce haploid eggs containing only the *H*. *octogrammus* genome (maternal ancestor) and generate diploid hemiclonal offspring by fertilization with haploid sperm from either *H*. *agrammus* or *H*. *otakii* (paternal species) [19]. Mating experiments in *Hexagrammos* hybrids have shown the existence of certain genetic factors that induce hybridogenesis [19]. Therefore, the hemiclonal Hoc* genome of the unisexual hybrids is differentiated from the *Hoc* genome of the pure species, and the two hemiclonal hybrids are denoted as Hoc*/*Hag* and Hoc*/*Hot* (“*” indicates that the genetic factors required for inducing hybridogenesis are present). Hoc*/*Hoc* (hereafter BC-Hoc*) individuals, which are produced by maternal backcrossing of Hoc*/*Hag* hybrids with *Hoc* individuals, have been shown to produce recombinant gametes despite having inherited a Hoc* genome from a hemiclonal hybrid (Fig 1) [23]. This suggests that the hemiclonal genes are not expressed between homologous genomes, but only when heterologous genomes are mated.

In our previous study, we elucidated a mechanism for reducing extinction risk by two-way backcrossing in the field [20]. The "recombination generation" produced by two-way backcrossing has been suggested to be important for overcoming the evolutionary dead-end of the hemiclonal lineage. Recombinant generations which carried hemiclonal genes in the gene pool of the maternal species would behave as *Hoc* in wild and seemed to be morphologically undistinguishable from the maternal species. Here, we examined whether BC-Hoc* and the new hybrid lineages produced from BC-Hoc* were not morphologically different to the *Hoc* and hybrids in wild populations. Finally, we try to produce a new hemiclonal lineage from BC-Hoc* that we generated by artificial fertilization to experimentally confirm that multiple genetic factors are implicated in expression of hybridogenesis and that evolutionary dead-ends can be overcome by shuffling the maternal genome (Fig 1).

## Materials and methods

### Fish sampling and artificial fertilization

The two *Hexagrammos* species (*Hoc* and *Hag*) and the natural hybrid (Hoc*/Hag) were caught by traps and/or fishing rods in the vicinity of the Usujiri Fisheries Station (N41°57’, E140°58’) of the Field Science Center for Northern Biosphere, Hokkaido University in southern Hokkaido, Japan, from 2010 to 2016. Until artificial fertilization, the captured fishes were kept in 500 L tanks containing concrete blocks which served as hiding places. The tanks were circulated with fresh seawater that was the same temperature as that of the natural environment. The fishes were fed daily with a diet consisting of Japanese anchovy, krill and artificial pellets (Otohime, Nishinmarubeni Co., Japan). In 2010, to produce BC-Hoc*, mature eggs from Hoc*/*Hag* hybrids were artificially fertilized with sperm from *Hoc* individuals. After two years (2012), when the BC-Hoc* individuals were mature, mature eggs of BC-Hoc* females were fertilized artificially with sperm from *Hag* individuals to produce BC-Hoc* × *Hag* individuals (A × B indicates A: female × B: male artificial crossing). By 2016, a total of 21 mature BC-Hoc* × *Hag* individuals (16 females and 5 males) had been raised. To confirm whether BC-Hoc* × *Hag* produced hemiclonal or recombined eggs, eggs from 16 BC-Hoc* × *Hag* females were fertilized with sperm from *Hag* or *Hoc* males. A total of seven larvae from each of the 16 clutches were then subjected to microsatellite DNA analysis.

Artificial fertilization was performed as described previously [22, 23] and the clutches were incubated for 20–23 days at 11–15°C until hatching. Tissue samples of hatched larvae and parental fishes for genetic analysis were preserved in 99% ethanol at -20°C. Samples of hatched larvae for genetic analysis were anesthetized using ethyl m-aminobenzoate methanesulfonate (Nacalai Tesque Inc., Kyoto, Japan) and fixed. Parental fishes used for artificial fertilization were housed for use in further research. Naturally dead BC-Hoc* and BC-Hoc* × *Hag* hybrids were fixed in 10% seawater formalin and preserved in 50% 2-propanol for morphological analysis.

### Ethics statement

The experimental procedures involving fish were approved by the Institutional Animal Care and Use Committee (The Regulations of Animal Experimentation at Hokkaido University), Permit number 26–1, according with directives from the Ministry of Education, Culture, Sports, Science and Technology, Japan.

### Morphological analysis

To ascertain the morphological identity of the BC-Hoc* to *Hoc* and BC-Hoc* × *Hag* to Hoc*/*Hag* hybrids, we counted seven parameters and measured nine parameters for morphological characters (listed in Table 1) for BC-Hoc* and BC-Hoc* × *Hag* individuals preserved in 2-propanol. Transversal line scales were counted only in BC-Hoc*. Morphometric data from 10 adult BC-Hoc* and 8 BC-Hoc* × Hag individuals were compared to those from 37 *Hoc*, 13 *Hag* and 4 Hoc*/*Hag* individuals by referring to [19]. To visualize the differences among the five lineages, the principal component analysis (PCA) was performed using five counts, excluding Transversal line scales and Lateral lines, and nine measurements converted to percentages of the standard length. Counts, measurements, and descriptive terminology followed [19, 24]. Since the tips of the fins of the fish bred in captivity showed signs of wear due to abrasion with the tank, and since no interspecific (lineage) differences in the length of each fin were observed in previous studies [19], fin length was not used as a morphometric character in this study. The principal Component Analysis was performed using R version 4.2.2 (R Core Team 2020).

**Table 1:** Counts and proportional morphometric characters used in this study.

	*Hag*	*Hoc*	BC-Hoc*	Hoc*/*Hag*	BC-Hoc* × *Hag*
	n = 13	n = 37	n = 5	n = 4	n = 8
Standard length (SL; mm)	144–262	117–246	122.5–159.96	219–255	165.7–200.8
Counts	Range				
Dorsal fin spines	17–19	18–22	18–19	18–19	17–18
Dorsal fin rays	20–22	22–25	21–24	20–23	21–23
Pectoral fin rays	16–18	17–19	18–19	17–19	18–19
Anal fin rays	18–21	23–26	23–25	21–23	21–24
Lateral line scales	80–87	85–95	85–94	87–89	87–91
Transversal line scales	n.d.	7–9	7–10	n.d.	n.d.
Lateral lines	1	5 (4.5)	5	Partial	Partial
Proportion in SL (%)					
Body depth	23.6–30.3	21.0–33.4	23.9–31.2	23.2–29.3	24.9–29.5
Caudal peduncle depth	9.5–11.7	8.3–10.8	8.4–10.3	9.6–10.3	9.3–11.2
pre dorsal fin length	27.0–29.6	24.4–29.0	25.9–26.9	25.0–28.4	26.3–29.1
Snout length	8.1–10.4	7.8–10.2	8.6–9.5	8.2–10.0	9.3–10.8
Upper jaw length	8.3–10.4	7.9–11.5	8.3–9.7	9.1–10.2	9.1–9.9
Dorsal base length	58.9–65.9	60.0–68.4	60.2–63.0	63.7–66.2	59.9–68.4
Anal base length	27.7–32.3	31.1–36.3	31.1–36.7	30.5–31.6	31.6–35.9
Caudal peduncle length	12.6–15.7	11.1–13.8	12.3–15.4	13.0–13.9	13.4–15.3
Interorbital width	5.0–6.1	4.7–6.1	4.7–6.4	5.0–5.4	5.5–6.1

Measurement data are given in percent standard length and presented as the range.

Count and measurement data for *Hag*, *Hoc* and Hoc*/*Hag* referred to data set of individuals without missing values used in [19].

Lateral line of *Hoc* is rarely disturbed by damage, etc.

### Detection of hemiclonal hybrids by microsatellite DNA

Seven microsatellite loci identified as informative in previous studies [19, 20, 25] were used to clarify the inheritance pattern of the gametes that BC-Hoc* × Hag hybrids produced (S1 Table). First, to clarify the genesis mode of each clutch, one primer sets for microsatellite loci were selected by focusing on loci where the parental fish exhibited heterozygosity. If two maternal alleles appeared alternatively in a clutch, then the BC-Hoc* × *Hag* hybrids was considered to have produced a recombinant gamete, and the analysis of such clutches was terminated. In all primer sets, if all offspring shared only a single maternal allele, then the BC-Hoc* × *Hag* hybrids was considered to have hybridogenesis.

Total genomic DNA was extracted using a Quick Gene DNA tissue kit S (Fujifilm, Japan) according to the manufacturer’s instructions and stored in a refrigerator at 4°C until use. The PCR mixes contained 12.5 μl of EmeraldAmp PCR Master Mix (Takara Bio Inc., Japan), 0.25 μl of each primer (20 μM), 1 μl of template DNA (50–100 ng/μl), and 11 μl of water to give a final volume of 25 μl. The PCR profiles for the seven regions were the same as those reported previously [20, 25]. Genotyping was performed by electrophoresis on 8.25% polyacrylamide gels stained with SYBR Green II (Takara Bio Inc., Japan).

## Results

### Morphological analysis

**Fig 2:**
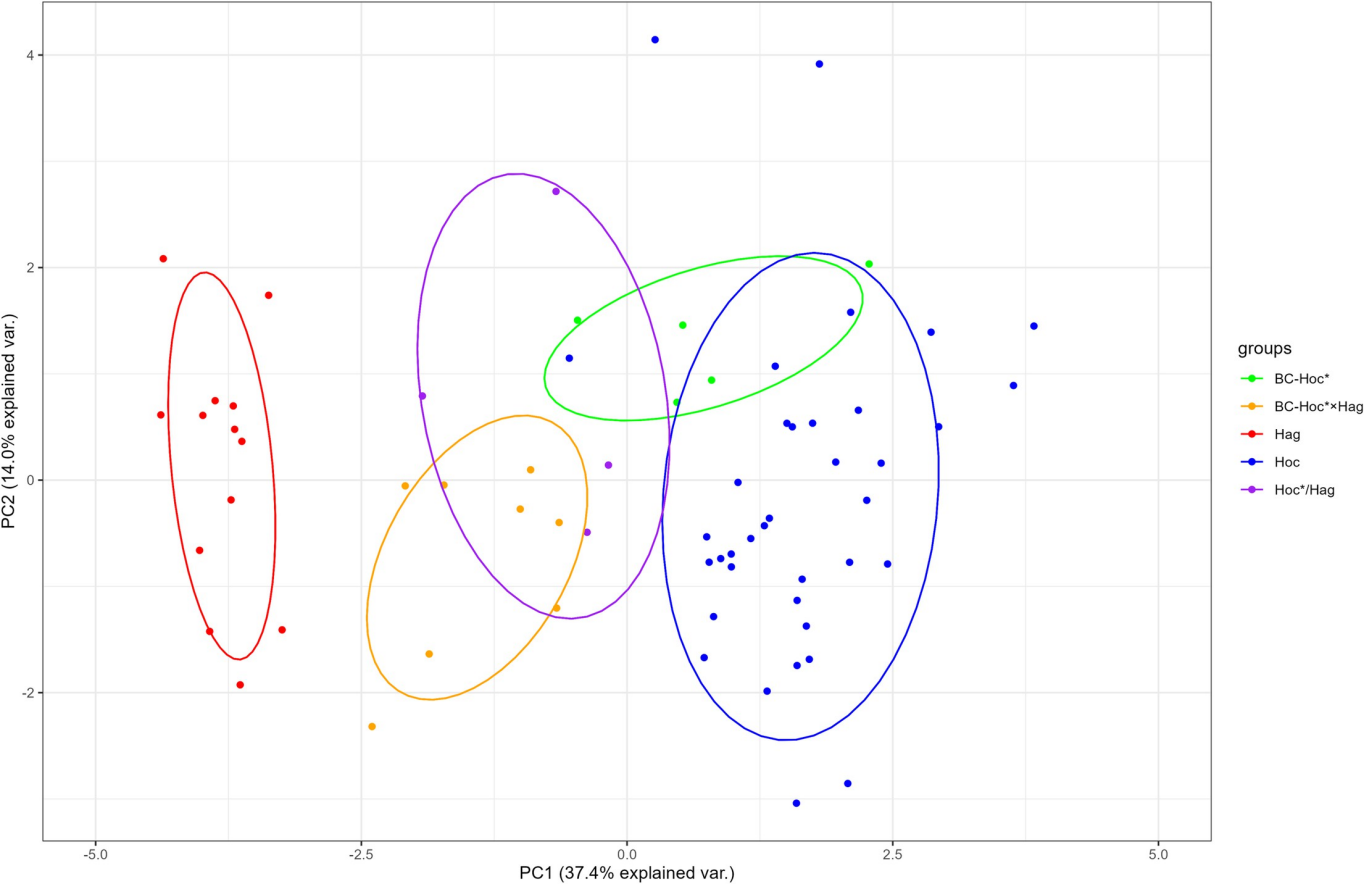
Scatterplot of the principal component analysis by 14 morphological characters of the 5 lineages used in this study. *Hoc*, BC-Hoc* and *Hag* indicate *H*. *octogrammus*, maternal backcross and *H*. *agrammus*, respectively; BC-Hoc*×*Hag* and Hoc*/*Hag* indicate hybrid lineages.

In the principal component analysis (PCA), the first, second and third PCs accounted for 37.4, 14.0 and 11.8% of the variance. *Hoc* and BC- Hoc*, as well as Hoc*/*Hag* and BC- Hoc* × Hag, formed distinct clusters with overlap; *Hoc* and *Hag* formed separate clusters, with hybrids (Hoc*/*Hag* and BC- Hoc* × Hoc) showing intermediate characters between two parental species (Fig 2).

Morphometric counts and measurements are given in Table 1 and S2 Table. On comparison of BC-Hoc* and *Hoc*, the ranges obtained for all counts overlapped. Both BC-Hoc* and *Hoc* individuals had five lateral lines. These results show that there is no morphological difference between *Hoc* and BC-Hoc*.

On comparison of BC-Hoc* × *Hag* and Hoc*/*Hag*, the ranges obtained for all counts and measurements overlapped. BC-Hoc* × *Hag* as well as Hoc*/*Hag* had partial first, second, fourth, and fifth lateral lines, indicating that BC-Hoc* × *Hag* individuals were morphologically indistinguishable to Hoc*/*Hag* individuals.

### Detection of hemiclonal hybrids

To detect heterozygosity, 109 offspring of the 16 BC-Hoc* × *Hag* hybrids were genotyped using microsatellite loci. The resulting inheritance patterns exhibited by the progeny of the 16 BC-Hoc* × Hag hybrids are shown in Tables 2 and 3. In one out of 16 clutches produced by the BC-Hoc* × *Hag* hybrids (i.e., 1/16: 6.25%), all of the resulting progeny shared the same maternal alleles for the 7 microsatellite loci examined (Table 2). This finding suggests that the haploid gametes were inherited hemiclonally in this clutch. In the remaining 15 clutches, either of the two maternal alleles were found alternatively in the resulting progeny, suggesting a recombinant mode of gamete production (Table 3).

**Table 2:** Genotypes of offspring from hemiclonal BC-*Hoc* × *Hag* by artificial fertilization experiments.

Clutch No.	Individual No.	Genotypes of microsatellite DNA													
		*Hexoc 6*		*Hexoc 14*		*Hexoc 21*		*Hexoc 2*		*Hexoc 9–2*		*Otakii 5*		*Otakii 6*	
6	Mother	194	128	130	88	146	126	94	88	148	142	138	147	154	140
	Offspring1	194	*128*	130	*94*	146	*124*	94	*88*	148	*130*	138	*142*	154	*120*
	Offspring2	194	*128*	130	*94*	146	*124*	94	*78*	148	*130*	138	*142*	154	*120*
	Offspring3	194	*128*	130	*86*	146	*124*	94	*88*	148	*126*	138	*148*	154	*132*
	Offspring4	194	*118*	130	*86*	146	*116*	94	*78*	148	*130*	138	*142*	154	*132*
	Offspring5	194	*118*	130	*86*	146	*124*	94	*88*	148	*130*	138	*142*	154	*120*
	Offspring6	194	*118*	130	*94*	146	*124*	94	*88*	148	*126*	138	*142*	154	*132*
	Offspring7	194	*118*	130	*86*	146	116	94	*78*	148	*126*	138	*148*	154	*120*
	Father	*128*	*118*	*94*	*86*	*124*	*116*	*88*	*78*	*130*	*126*	*148*	*142*	*132*	*120*

Blue highlight indicates alleles showing hemiclonal maternal inheritance.

Italics indicated alleles inherited from the father.

**Table 3:** Genotypes of offspring from recominant BC-*Hoc* × *Hag* by artificial fertilization experiments.

Clutch No.	Individual No.	*Hexoc 6*		Clutch No.	Individual No.	*Hexoc 14*		Clutch No.	Individual No.	*Hexoc 21*	
2	mother	118	106	1	mother	128	92	7	mother	150	100
	Offspring1	118	126		Offspring1	92	86		Offspring1	150	124
	Offspring2	118	124		Offspring2	128	86		Offspring2	100	124
	Offspring3	118	126		Offspring3	92	86		Offspring3	100	124
	Offspring4	106	126		Offspring4	128	88		Offspring4	100	124
	Offspring5	106	126		Offspring5	92	88		Offspring5	100	116
	Offspring6	118	126		Offspring6	92	86		Offspring6	150	116
	Offspring7	106	124		Offspring7	128	88		Offspring7	100	124
	father	126	124		father	88	86		father	124	116
9	mother	126	106	3	mother	130	92	10	mother	140	124
	Offspring1	106	116		Offspring1	92	88		Offspring1	140	124
	Offspring2	n.d.	n.d.		Offspring2	130	86		Offspring2	124	124
	Offspring3	106	116		Offspring3	130	88		Offspring3	140	116
	Offspring4	126	126		Offspring4	130	86		Offspring4	140	116
	Offspring5	126	126		Offspring5	92	86		Offspring5	140	124
	Offspring6	106	116		Offspring6	92	88		Offspring6	140	116
	Offspring7	106	116		Offspring7	130	88		Offspring7	124	116
	father	126	116		father	88	86		father	124	116
				4	mother	130	92	12	mother	140	124
					Offspring1	130	92		Offspring1	140	116
					Offspring2	130	92		Offspring2	124	124
					Offspring3	130	92		Offspring3	124	124
					Offspring4	130	88		Offspring4	140	124
					Offspring5	130	88		Offspring5	124	124
					Offspring6	92	88		Offspring6	124	116
					Offspring7	92	92		Offspring7	140	116
					father	92	88		father	124	116
				5	mother	130	92	14	mother	150	124
					Offspring1	130	92		Offspring1	150	124
					Offspring2	92	88		Offspring2	150	116
					Offspring3	92	88		Offspring3	150	124
					Offspring4	92	92		Offspring4	124	116
					Offspring5	130	92		Offspring5	150	116
					Offspring6	92	92		Offspring6	124	124
					Offspring7	130	88		Offspring7	150	124
					father	92	88		father	124	116
				8	mother	130	94	15	mother	146	100
					Offspring1	94	86		Offspring1	146	116
					Offspring2	94	94		Offspring2	100	124
					Offspring3	130	94		Offspring3	100	124
					Offspring4	94	94		Offspring4	146	116
					Offspring5	130	94		Offspring5	100	116
					Offspring6	94	86		Offspring6	100	116
					Offspring7	94	86		Offspring7	100	116
					father	94	86		father	124	116
				11	mother	128	94				
					Offspring1	n.d.	n.d.				
					Offspring2	94	86				
					Offspring3	128	86				
					Offspring4	94	86				
					Offspring5	128	86				
					Offspring6	94	86				
					Offspring7	94	94				
					father	94	86				
				13	mother	128	88				
					Offspring1	128	86				
					Offspring2	128	94				
					Offspring3	n.d	n.d				
					Offspring4	128	86				
					Offspring5	128	94				
					Offspring6	128	94				
					Offspring7	88	86				
					father	94	86				
				16	mother	130	92				
					Offspring1	130	130				
					Offspring2	130	130				
					Offspring3	92	124				
					Offspring4	130	124				
					Offspring5	130	130				
					Offspring6	92	124				
					Offspring7	130	124				
					father	130	124				

Blue—Green and Red—Orange indicated alleles inherited from the mother and father, respectively. Alleles present in both parents are shown in grey.

## Discussion

### Hybridogenesis expressed through genomic dissimilarity and genetic factors

Genomic heterogeneity (genetic affinity) between parental species plays an important role in the occurrence of hybridogenesis [17], but it is insufficient to induce this reproductive mode, which necessitates the conditional expression of certain genetic factors [23, 25]. The functionality of these unidentified genetic factors is hindered by the high genetic affinity observed between homologous chromosomes. This is substantiated by the observation that BC-Hoc*, comprising Hoc*/*Hoc*, produced recombined gametes through normal meiosis [19, 23]. That is to say, BC-Hoc* individuals can be characterized as carriers of hemiclonal genes.

**Fig 3:**
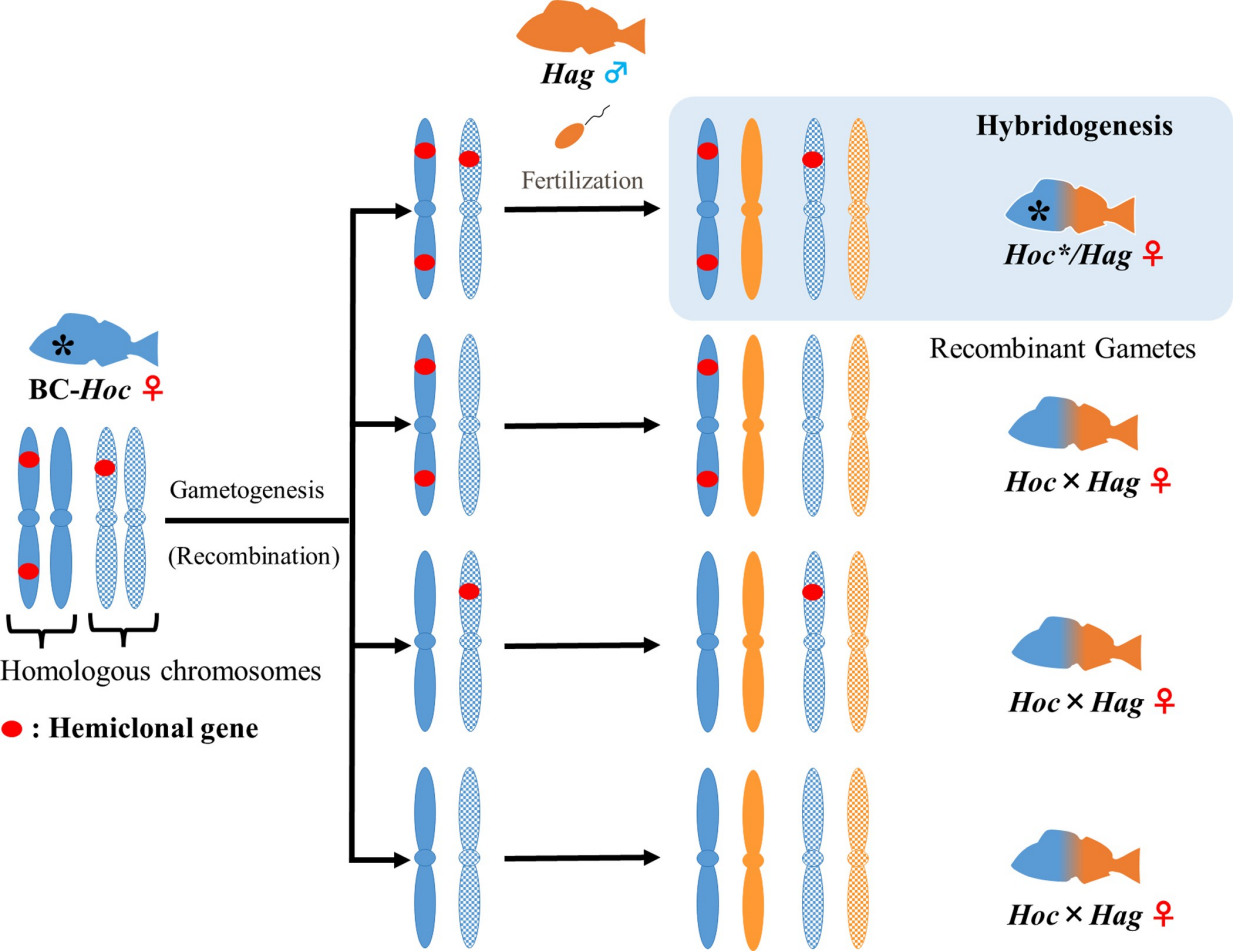
Inheritance pattern of recombination in three hemiclonal genes during gametogenesis. During gametogenesis of BC- Hoc*, chromosomes containing multiple hemiclonal genes (solid red circles) inherited from Hoc*/*Hag* recombine with homologous chromosomes inherited from *Hoc*. If all randomly rearranged hemiclonal genes are inherited, then new hemiclonal lineages are regenerated. Homologous chromosomes are comprised of either two solid or two stippled chromosomes. *Hoc* and *Hag* represent *H*. *octogrammus* and *H*.*agrammus*, respectively. Blue and orange indicate *H*. *octogrammus* and *H*. *agrammus*, respectively.

In the present study, hybrids (BC-Hoc* × Hag) of the recombinant generation (BC-Hoc*) and *Hag* have shown a 1/16 (6.25%) chance of undergoing hybridogenesis. In eggs produced by BC-Hoc*, the genetic factor responsible for inducing hybridogenesis would be inherited by random recombination. If this factor was encoded by a single gene, then approximately half of BC-Hoc* × Hag should mature into hybridogens. The marked discrepancy between the observed result and the initial prediction, the result indicates that the genetic factor responsible for inducing hybridogenesis involves multiple genes, which are located at sites that are susceptible to recombination (i.e., either at distant positions on the same chromosome or on different chromosomes) (Fig 3). Gametogenesis by hybridogenesis requires several extraordinary steps, namely elimination of the paternal genome and duplication of the maternal genome [10, 26–29]. Although the specific genes that are associated with hybridogenesis were not identified in this study, it is suggested that hemiclonal genes regulate several processes, including recognition and elimination of the paternal genome and selective preservation of the maternal genome.

In the hybrids *Poeciliopsis monacha-occidentalis* and *P*. *monachal-lucida*, the observed rates of hemiclonal hybrids resulting from artificial crosses between parental species were also low at 30% and 7%, respectively, suggesting that reestablishing hybridogenesis in these species is also difficult [30]. Consequently, it is considered that the genetic factors that induce hybridogenesis are present in other cases of hybridogenesis, as well as in the genus *Hexagrammos*. The genus *Bacillus* (stick insect) hybrids, *Pelophylax* (*Rana*) *esculenta* (frog) and *Misgurnus anguillicaudatus* (loaches) have been reported to have various reproductive modes [4, 31, 32]. In these lineages also, more complex mechanisms may be required to determine the reproductive mode.

### Longevity and genetic diversity in hemiclonal organisms

The present study employed artificial crosses to demonstrate that a new hemiclone lineage can arise through recombinant generations (BC-Hoc*). Genetic diversity is unlikely to increase in the hemiclonal lineages and deleterious mutations are expected to accumulate if hybridogens are persistently backcrossed with males of the paternal species. This accumulation of deleterious mutations can be mitigated through the emergence of a new hemiclonal lineage through the BC-Hoc* generation, which produces recombined gametes. The offspring between *P*. *monacha*-*lucida* hemiclonal hybrids, which inherit only *P*. *monacha* genes, and *P*. *viriosa*, a sympatric sister species to *P*. *monacha*, produce recombinant gametes [33, 34]. In addition, it has been suggested that *P*. *monacha*-*lucida* hybrids may have obtained genetic variation by backcrossing with *P*. *monacha* [35]. That is, backcrossing between Hoc*/*Hag* and *Hoc* males could also potentially play an important role in increasing the genetic diversity of the hemiclonal lineage. Indeed, the occurrence of BC-Hoc* by natural mating between Hoc*/*Hag* and *Hoc* males has been inferred using a specific cytogenetic marker, with Hoc*/*Hag* individuals observed to backcross with *Hag* and *Hoc* individuals with almost even probability [20]. In hybrids that employ (hemi)clonal reproduction, changes in the species of the sperm donor are referred to as “host switching” [25, 36], and the mate choice among such hybrids is referred to as "two-way backcrossing (crossing of hemiclonal hybrids with males of both parental species)" [20]. This study showed that BC- Hoc* (carrier) individuals cannot be morphologically identified to *Hoc* individuals. These results suggest that BC-Hoc* fits successfully into the *Hoc* population and that the Hoc* genome is almost certainly present within the *Hoc* gene pool in the wild.

Regeneration of new hemiclonal lineages is arisen from the *Hoc* gene pool when a hybrid possesses specific set of genetic factors that induce hybridogenesis. It is expected that distinct variations from the previous hemiclonal lineage will exist within the regenerated hemiclonal lineage that experience one or more recombinant generations. Given that the gene set is hypothesized to be comprised of several diverse components, regeneration is expected to occur only rarely. Therefore, when hybridogenesis reoccurs, the lineage will retain renewed genetic diversity. The BC-Hoc* × Hag hybrids cannot be identified as hemiclonal hybrids (Hoc*/*Hag*), indicating that the hybrids produced by the regeneration event have fitted into the wild hybrid population. This interpretation is supported by the previous reports. Hoc*/*Hag* hybrids possess polyphyletic haplotypes in mtDNA genealogical tree [25], indicating multiple origins. Estimates of genetic diversity based on mtDNA haplotypes showed that the regeneration events described in the present study occurred several times over the last 2.2–3.6 million years, i.e., after contact between populations of *Hoc* and *Hag* [22, 25].

A similar diversity of mtDNA haplotypes has been observed in extant hemiclonal hybrids of *P*. *monachal-lucida* (e.g., [37]). In *P*. *esculentus*, in which hemiclonal males have also been reported, mtDNA exchange between parental species has been observed through backcrosses [38, 39]. Although classification of the genetic factors responsible for inducing hemiclones has yet to be elucidated in these taxa, the regeneration of new hemiclonal lineages through recombining generations, like BC-*Hoc*, could be one of the major keys to evolutionary success.

## Supporting information

S1 Table Primers used in this study. (XLSX)

S2 Table Counts and proportional morphometric characters. In *Hoc* and BC-Hoc*, scales on the transversal line between the second and third lateral lines were counted.
(XLSX)

## References

[1] Scribner KT, Page KS, Bartron ML. Hybridization in freshwater fishes: a review of case studies and cytonuclear methods of biological inference. Rev Fish Biol Fish. 2000; 10(3):293–323. doi: 10.1023/A:1016642723238

[2] Lamatsch DK, Stöck M. Sperm-Dependent Parthenogenesis and Hybridogenesis in Teleost Fishes. In: Schön I, Martens K, Dijk P, editors. Lost Sex: The Evolutionary Biology of Parthenogenesis. Dordrecht: Springer Netherlands; 2009. p. 399–432. doi: 10.1007/978-90-481-2770-2_19

[3] Lampert KP, Schartl M. The origin and evolution of a unisexual hybrid: Poecilia formosa. Philos Trans R Soc Lond B Biol Sci. 2008; 363(1505):2901–9. doi: 10.1098/rstb.2008.0040 18508756 PMC2606734

[4] Morishima K, Yoshikawa H, Arai K. Meiotic hybridogenesis in triploid Misgurnus loach derived from a clonal lineage. Heredity. 2008; 100(6):581–6. doi: 10.1038/hdy.2008.17 18382473

[5] Dawley RM. An introduction to unisexual vertebrates. In: Dawley RM, Bogart JP, editors. Evolution and Ecology of Unisexual Vertebrates. New York State Museum; 1989. p. 1–18.

[6] Vrijenhoek RC. A list of known unisexual vertebrates. In: Dawley RM, Bogart JP, editors. Evolution and ecology of unisexual vertebrates. New York: New York State Museum; 1989. p. 19–23.

[7] Burt A, Trivers R. Genome exclusion. In: Burt A, Trivers R, editors. Genes in Conflict. Cambridge: Harvard University Press; 2009. p. 381–419. doi: 10.4159/9780674029118/html

[8] Schultz RJ. Reproductive Mechanism of Unisexual and Bisexual Strains of the Viviparous Fish Poeciliopsis. Evolution. 1961;15(3):302–25.

[9] Cimino MC. Egg-production, polyploidization and evolution in a diploid all-female fish of the genus Poeciliopsis. Evolution. 1972; 26(2):294–306. doi: 10.1111/j.1558-5646.1972.tb00195.x 28555744

[10] Tunner HG, Heppich-Tunner S. Genome exclusion and two strategies of chromosome duplication in oogenesis of a hybrid frog. Naturwissenschaften. 1991;78:32–4.

[11] Lynch M, Gabriel W. MUTATION LOAD AND THE SURVIVAL OF SMALL POPULATIONS. Evolution. 1990; 44(7):1725–37. doi: 10.1111/j.1558-5646.1990.tb05244.x 28567811

[12] Lynch M, Bürger R, Butcher D, Gabriel W. The mutational meltdown in asexual populations. J Hered. 1993; 84(5):339–44. doi: 10.1093/oxfordjournals.jhered.a111354 8409355

[13] Loewe L, Lamatsch DK. Quantifying the threat of extinction from Muller’s ratchet in the diploid Amazon molly (Poecilia formosa). BMC Evol Biol. 2008; 8:88. doi: 10.1186/1471-2148-8-88 18366680 PMC2292145

[14] Schartl M, Nanda I, Schlupp I, Wilde B, Epplen JT, Schmid M, et al. Incorporation of subgenomic amounts of DNA as compensation for mutational load in a gynogenetic fish. Nature. 1995; 373(6509):68–71.

[15] Freitas SN, Harris DJ, Sillero N, Arakelyan M, Butlin RK, Carretero MA. The role of hybridisation in the origin and evolutionary persistence of vertebrate parthenogens: a case study of Darevskia lizards. Heredity. 2019; 123(6):809–10. doi: 10.1038/s41437-019-0270-7 31413332 PMC6834615

[16] Wetherington JD, Kotora KE, Vrijenhoek RC. A TEST OF THE SPONTANEOUS HETEROSIS HYPOTHESIS FOR UNISEXUAL VERTEBRATES. Evolution. 1987; 41(4):721–31. doi: 10.1111/j.1558-5646.1987.tb05848.x 28564354

[17] Moritz C, Brown WM. Genetic diversity and the dynamics of hybrid parthenogenesis in Cnemidophorus (Teiidae) and Heteronotia (Gekkonidae). In: Dawley RM, Bogart JP, editors. Evolution and ecology of unisexual vertebrates. New York: New York State Museum; 1989. p. 87–112.

[18] Murphy RW, Fu J, Macculloch RD, Darevsky IS, Kupriyanova LA. A fine line between sex and unisexuality: the phylogenetic constraints on parthenogenesis in lacertid lizards. Zool J Linn Soc. 2000;130(4):527–49.

[19] Kimura-Kawaguchi MR, Horita M, Abe S, Arai K, Kawata M, Munehara H. Identification of hemiclonal reproduction in three species of Hexagrammos marine reef fishes. J Fish Biol. 2014;85(2):189–209. doi: 10.1111/jfb.12414 24903212

[20] Suzuki S, Miyake S, Arai K, Munehara H. Unisexual hybrids break through an evolutionary dead end by two-way backcrossing. Evolution. 2020;74(2):392–403. doi: 10.1111/evo.13903 31873961

[21] Rutenberg EP. Survey of the fishes of family Hexagrammidae. In: Rass TS, editor. Greenlings: Taxonomy, Biology and Interoceanic Transplantation. Jerusalem: Israel Program for Scientific Translations; 1970. p. 1–103.

[22] Crow KD, Munehara H, Bernardi G. Sympatric speciation in a genus of marine reef fishes. Mol Ecol. 2010; 19(10):2089–105. doi: 10.1111/j.1365-294X.2010.04611.x 20345669

[23] Suzuki S, Arai K, Munehara H. Karyological evidence of hybridogenesis in Greenlings (Teleostei: Hexagrammidae). PLoS One. 2017; 12(7):e0180626. doi: 10.1371/journal.pone.0180626 28678883 PMC5498075

[24] Hubbs CL, Lagler KF. Fishes of the great lakes region. Bull Cranbrook Inst Sci. 1958; 26:1–213.

[25] Munehara H, Horita M, Kimura-Kawaguchi MR, Yamazaki A. Origins of two hemiclonal hybrids among three Hexagrammos species (Teleostei: Hexagrammidae): genetic diversification through host switching. Ecol Evol. 2016; 6(19):7126–40. doi: 10.1002/ece3.2446 28725387 PMC5513241

[26] Vinogradov AE, Borkin LJ, Günther R, Rosanov JM. Genome elimination in diploid and triploid Rana esculenta males: cytological evidence from DNA flow cytometry. Genome. 1990; 33(5):619–27. doi: 10.1139/g90-092 2262136

[27] Ogielska M. Nucleus-like bodies in gonial cells of Rana esculenta [Amphibia, Anura] tadpoles-a putative way of chromosome elimination. Zool Pol. 1994;39(3–4):461–74.

[28] Chapter Ogielska M. 8 Development and Reproduction of Amphibian Species, Hybrids, and Polyploids. In: Ogielska M, editor. Reproduction of amphibians. Boca raton, FL: CRC Press; 2009. p. 343–410.

[29] Majtánová Z, Dedukh D, Choleva L, Adams M, Ráb P, Unmack PJ, et al. Uniparental genome elimination in Australian carp gudgeons. Genome Biol Evol. 202; doi: 10.1093/gbe/evab030 33591327 PMC8245195

[30] Schultz RJ. Unisexual Fish: Laboratory Synthesis of a “Species.” Science. 1973;179(4069):180–1. doi: 10.1126/science.179.4069.180 4682248

[31] Mantovani B, Scali V. HYBRIDOGENESIS AND ANDROGENESIS IN THE STICK-INSECT BACILLUS ROSSIUS‐GRANDII BENAZZII (INSECTA, PHASMATODEA). Evolution. 1992 Jun; 46(3):783–96. doi: 10.1111/j.1558-5646.1992.tb02084.x 28568678

[32] Pruvost NBM, Hoffmann A, Reyer H-U. Gamete production patterns, ploidy, and population genetics reveal evolutionary significant units in hybrid water frogs (Pelophylax esculentus). Ecol Evol. 2013; 3(9):2933–46. doi: 10.1002/ece3.687 24101984 PMC3790541

[33] Vrijenhoek RC, Schultz RJ. EVOLUTION OF A TRIHYBRID UNISEXUAL FISH (POECILIOPSIS, POECILIIDAE). Evolution. 1974; 28(2):306–19. doi: 10.1111/j.1558-5646.1974.tb00750.x 28563275

[34] Mateos M, Vrijenhoek RC. Ancient versus reticulate origin of a hemiclonal lineage. Evolution. 2002; 56(5):985–92. doi: 10.1111/j.0014-3820.2002.tb01410.x 12093033

[35] Vrijenhoek RC. Genetics of a Sexually Reproducing Fish in a Highly Fluctuating Environment. Am Nat. 1979; 113(1):17–29. doi: 10.1086/283362

[36] Choleva L, Apostolou A, Rab P, Janko K. Making it on their own: sperm-dependent hybrid fishes (Cobitis) switch the sexual hosts and expand beyond the ranges of their original sperm donors. Philos Trans R Soc Lond B Biol Sci. 2008; 363(1505):2911–9. doi: 10.1098/rstb.2008.0059 18508748 PMC2606746

[37] Quattro JM, Avise JC, Vrijenhoek RC. Molecular evidence for multiple origins of hybridogenetic fish clones (Poeciliidae:Poeciliopsis). Genetics. 1991; 127(2):391–8. doi: 10.1093/genetics/127.2.391 2004710 PMC1204366

[38] Spolsky C, Uzzell T. Evolutionary history of the hybridogenetic hybrid frog Rana esculenta as deduced from mtDNA analyses. Mol Biol Evol. 1986; 3(1):44–56. doi: 10.1093/oxfordjournals.molbev.a040376 2832687

[39] Holsbeek G, Jooris R. Potential impact of genome exclusion by alien species in the hybridogenetic water frogs (Pelophylax esculentus complex). Biol Invasions. 2009; 12(1):1. doi: 10.1007/s10530-009-9427-2

